# Age, gender, and puberty influence the development of facial emotion recognition

**DOI:** 10.3389/fpsyg.2015.00761

**Published:** 2015-06-16

**Authors:** Kate Lawrence, Ruth Campbell, David Skuse

**Affiliations:** ^1^Department of Psychology, St Mary’s University, Twickenham, LondonUK; ^2^Deafness Cognition and Language Centre, University College LondonLondon, UK; ^3^Behavioural and Brain Sciences Unit, UCL Institute of Child Health, LondonUK

**Keywords:** emotion, social cognition, facial expression, emotion recognition, child development, face recognition

## Abstract

Our ability to differentiate between simple facial expressions of emotion develops between infancy and early adulthood, yet few studies have explored the developmental trajectory of emotion recognition using a single methodology across a wide age-range. We investigated the development of emotion recognition abilities through childhood and adolescence, testing the hypothesis that children’s ability to recognize simple emotions is modulated by chronological age, pubertal stage and gender. In order to establish norms, we assessed 478 children aged 6–16 years, using the Ekman-Friesen Pictures of Facial Affect. We then modeled these cross-sectional data in terms of competence in accurate recognition of the six emotions studied, when the positive correlation between emotion recognition and IQ was controlled. Significant linear trends were seen in children’s ability to recognize facial expressions of happiness, surprise, fear, and disgust; there was improvement with increasing age. In contrast, for sad and angry expressions there is little or no change in accuracy over the age range 6–16 years; near-adult levels of competence are established by middle-childhood. In a sampled subset, pubertal status influenced the ability to recognize facial expressions of disgust and anger; there was an increase in competence from mid to late puberty, which occurred independently of age. A small female advantage was found in the recognition of some facial expressions. The normative data provided in this study will aid clinicians and researchers in assessing the emotion recognition abilities of children and will facilitate the identification of abnormalities in a skill that is often impaired in neurodevelopmental disorders. If emotion recognition abilities are a good model with which to understand adolescent development, then these results could have implications for the education, mental health provision and legal treatment of teenagers.

## Introduction

Faces are of unrivaled significance to human social interactions. Not only do faces provide us with visual information that allows us to determine the sex, age, familiarity and identity of an individual, we also use faces to gather information about what other individuals might be thinking or feeling. Analysing and interpreting facial expressions of emotion is necessary to enable us to modify our social interactions appropriately. Over the decades since the 1970s, social and psychological research has established the universality of the six main facial expressions of emotion (Ekman and Friesen, 1971). Very young infants can discriminate some, but not all, facial expressions of emotion (Serrano et al., 1992; Peltola et al., 2008) and facial emotion recognition ability is impaired in numerous psychological disorders (Fairchild et al., 2009; Eussen et al., 2015; Evers et al., 2015; Maat et al., 2015; Mancuso et al., 2015; Taylor et al., 2015). However, our knowledge of the development of this ability throughout childhood and, in particular, adolescence is surprisingly sparse. This study aims to explore the quantitative and qualitative changes in facial emotion recognition accuracy across this period of development.

It has been suggested that, by 6 years of age, typically developing children are relatively accurate at discriminating several facial expressions of emotion (Izard, 1971), with some studies suggesting that near-adult levels of recognition are achieved before adolescence (Tremblay et al., 2001; Rodger et al., 2015). Other studies, however, suggest childhood difficulties in recognizing expressions of fear that persist into adolescence (Baird et al., 1999; Lenti et al., 1999). As a review by Herba and Phillips (2004) pointed out, no studies to date have examined the developmental trajectory of emotion recognition development throughout childhood and adolescence. Although there now exist a handful of reports focusing on this age range, the methodologies employed differ to a straightforward emotion recognition labeling paradigm (Thomas et al., 2007; Rodger et al., 2015) and the majority of studies focus their attention on the early childhood period, with few even considering 8–11 year olds (Gao and Maurer, 2010; Mancini et al., 2013 being notable exceptions).

Gao and Maurer (2010) used a paradigm which manipulated intensity of facial expression, comparing groups of children aged 5, 7, and 10 years with a group of adults. Children selected the appropriate emotion from a choice of four in two separate sets (either neutral, happy, surprised, scared; or neutral, sad, angry, disgusted). The intensity of the emotion displayed varied between 10 and 100%, with the threshold for emotion recognition calculated. Sensitivity to happy facial expressions was at adult levels in children as young as 5 years of age, but for the other emotions there was some increase in sensitivity between the youngest children and adulthood. This raises some interesting questions with regard to the development of emotion recognition, including, ‘is improvement evident in adolescence?’ (there were no participants between 10 years of age and adulthood) and, ‘what would the results look like if participants had the six basic emotions to choose between?’ This final point may have a particular bearing on the results, since Gao and Maurer (2010) report near-ceiling levels of accuracy for recognition of the emotions when intensity reached about 50%, even in the youngest of viewers. Such levels are achieved only for happiness when task-demands required participants to choose between 6 emotion labels (Lawrence et al., 2003b).

A more recent study (Mancini et al., 2013) included a paradigm that called upon participants (aged between 8 and 11 years) to choose between the six basic emotions, together with neutral, for each face shown. Recognition accuracy increased during this period of mid-childhood, except (as found by Gao and Maurer, 2010) for happy faces. The largest age-related increases were noted for neutral and sad faces. Employing a complex paradigm to assess the perceptual threshold for detecting different emotional expressions, Rodger et al. (2015) found that sensitivity to emotional expressions increased from 5 years of age up until adulthood, for all expressions except those of happiness and fear. It would seem that the young children possessed adult-levels of sensitivity for detecting happiness and fear in faces. The stability of happiness recognition across this age range, and the fact that we are sensitive to this emotion from a young age is consistent with the findings of Mancini et al. (2013).

Looking directly at emotion recognition during late childhood (7–13 years), adolescence (14–18 years), and adulthood (25–57 years), Thomas et al. (2007) found that there was increased sensitivity to subtle changes in emotional expression in adults compared to the younger age groups. This study morphed faces between expressions of anger, fear, and neutrality, reporting linear trends in sensitivity to the changes throughout these three stages of life. Notably, for facial expressions morphed between neutral and anger, a quadratic trend was identified, whereby sensitivity to anger was equivalent in older children and adolescents but showed a marked increase between adolescence and adulthood. It is unknown whether the other basic emotions (not looked at in this study) would also continue to develop throughout adolescence.

From the above studies, we see suggestions of improvements in facial emotion recognition during childhood and adolescence. However, the different methodologies and age groups used, together with the differing emotions included, make it difficult to comprehensively understand the quantitative and qualitative developments in emotion recognition during this period of life. The current study sets out to systematically assess recognition accuracy for the six basic emotional expressions throughout childhood and adolescence. The primary aim of the current study was to explore the developmental trajectory of explicit facial emotion recognition in a large sample of children and adolescents split into large year-band categories, using original photographs from the Ekman-Friesen Pictures of Facial Affect (Ekman and Friesen, 1976).

The Ekman-Friesen Pictures of Facial affect test (Ekman and Friesen, 1976) has been used in hundreds of studies, over numerous decades, to assess the ability to recognize the six basic emotions within facial expressions; happiness, sadness, fear, surprise, disgust, and anger. The test consists of selecting which of these six emotions is best represented by each of a series of photographs. Ten images of each emotion (total 60 images) are shown in a random order and a mixture of male and female images are used. The test has been shown to have good reliability (Ekman and Friesen, 1976; Frank and Stennett, 2001), and also to be applicable for use with differing age groups from young children (Uljarevic and Hamilton, 2013), through to older adults (Calder et al., 2003). This test has not just furthered our academic understanding of emotion recognition, but is also used in clinical and educational settings to evaluate emotion recognition ability in children with developmental disorders and those with special educational needs.

In general, when emotion recognition is assessed in adult patients, the ability of a patient to recognize facial expressions is compared to standardized adult norms for such tests, but this is not possible for children since child norms do not exist. Although other tests have been developed in more recent years (including the use of morphed faces and those with cropped hairlines), very often in clinical and educational situations and for research with these groups these original Ekman faces are used as an assessment tool (Unoka et al., 2011; Cantalupo et al., 2013; Collin et al., 2013; Demirel et al., 2014; Gomez-Ibanez et al., 2014). There are differing merits to using different tests of emotion recognition. This study chose to use the basic set of faces for its ecological validity and also so that the normative data derived might be of use to clinical and educational psychologists using the original version of the task.

Several childhood neurodevelopmental disorders influence the ability to recognize facial expressions. There are difficulties recognizing facial expressions encountered by children with autism and Asperger syndrome (for example, Hobson et al., 1988; Howard et al., 2000; Sachse et al., 2014; Taylor et al., 2015). Children with psychopathic tendencies have also been reported to show selective impairments in the recognition of sad and fearful facial expressions of emotion (Stevens et al., 2001) which may extend to other emotions (Dawel et al., 2012). The lack of systematically gathered normative data on the ability of typically developing children to recognize facial expressions hampers our understanding of the nature and severity of such deficits. The correct interpretation of suspected impairments in childhood requires an understanding of the normal range of ability at any given age, and one aim of the current study was to establish such norms for children of school age. A newly developed facial identity recognition test for children [The Cambridge Face Memory Test for Children (CFMT-C)] presents norms for children from 5 to 12 years, shows a developmental improvement across this period, and is capable of detecting face recognition memory deficits in children with autism (Croydon et al., 2014). It is hoped that norms for the emotion recognition task could be equally successful in aiding the detection of deficits in children with atypical development. In order to understand the developmental normative data qualitatively, as well as quantitatively, the current study sets out to explore the effect of IQ, gender and puberty on emotion recognition ability.

Is the ability to recognize facial expressions of emotions associated with IQ? For children with autistic spectrum disorders and for psychiatric control children both verbal memory and Performance IQ have been found to predict emotion recognition ability (Buitelaar et al., 1999). Previous research by our group has shown that, within this same sample of children, recognition memory ability for facial identity was positively and significantly correlated with general cognitive ability (Lawrence et al., 2008). Another aim of the study was to assess the relationship between emotion recognition and IQ in typically developing children.

Both sexes are competent at recognizing facial expressions of emotion, with many studies finding that males and females perform at equivalent levels on a wide variety of emotion recognition tasks (Hall and Matsumoto, 2004). However, when differences are reported, they typically show a female advantage with women being more accurate decoders than men (Hall, 1978; Hall et al., 1999). One study, for example, found that females had a higher rate of correct classification of facial expressions, with males being more likely to have difficulty distinguishing one emotion from another (Thayer and Johnsen, 2000). This finding holds both for basic emotional expressions (Hall, 1978; McClure, 2000; Montagne et al., 2005; Biele and Grabowska, 2006; Mancini et al., 2013) and for more complex emotional and mental states (Baron-Cohen et al., 1997; Alaerts et al., 2011). Furthermore, males and females have been reported to show distinctive patterns of activation in neural regions involved in the processing of facial expressions of emotion including the amygdala and prefrontal cortex (Killgore et al., 2001), suggesting the possibility of different underlying mechanisms for processing them.

With respect to children specifically, Thomas et al. (2007) reported no sex differences in sensitivity to fearful and angry facial expressions, whilst other studies have suggested a constant female advantage during late childhood for facial emotion recognition (Montirosso et al., 2010). It is likely that any differences that do exist may be subtle, with research suggesting gender differentiated development of sadness and disgust recognition during childhood (Mancini et al., 2013) in the absence of differences in final levels of ability in the oldest children tested. It is unknown what happens to these developmental trends during adolescence. Not all studies of emotion recognition during childhood have explored sex differences (e.g., Gao and Maurer, 2010) and this is clearly an area that would benefit from greater exploration with a large sample across the childhood and adolescent period. One aim of the current study was to test for gender differences in the development of our ability to recognize different facial expressions of emotion across childhood and adolescence.

Given that recognition accuracy, even in young children, is relatively good, a question that needs addressing is whether there are qualitative changes in the development of facial expression recognition during childhood and adolescence and, if so, whether these changes themselves might influence recognition accuracy. In a very insightful review concerning the interplay between face recognition, adolescence and behavioral development, it is suggested that, as adolescents reorient away from their parents toward their peers, there is an increased drive for peer-acceptance and increased sensitivity to peer evaluation (Scherf et al., 2012). This may lead to qualitative changes in the type of information that is extracted from faces, with a greater emphasis than before being placed on appraisals of attractiveness and social status, together perhaps with greater sensitivity to displays of negative peer-evaluation. Scherf et al. (2012) suggest that, as a result of the changing way in which facial information is utilized, developmental differences in face processing abilities will emerge during this period of life. This notion is supported by the fact that facial attractiveness ratings undergo both quantitative and qualitative changes during late-childhood and early-adolescence (Cooper et al., 2006; Saxton et al., 2006). It has been suggested that the own-age bias in face recognition, representing superior recognition abilities for faces of a similar age to the viewer, may emerge as a result of social reorientation toward peers during this period of development (Scherf et al., 2012).

Is it possible that hormonal surges associated with puberty may influence the development of our ability to recognize facial expressions of emotion? There is evidence to suggest that hormonal fluctuations during the menstrual cycle influence fear recognition accuracy in females (Pearson and Lewis, 2005) and that emotion recognition abilities may be influenced by hormonal changes in late pregnancy (Pearson et al., 2009). Additional evidence that hormones may influence emotion recognition ability comes from studies of individuals who have Turner syndrome, a single X chromosome and are lacking in endogenous estrogen. Studies by our group have shown that these women have deficits in recognizing emotion and higher-order mental state information from facial expressions (Lawrence et al., 2003a,b). Furthermore, it has long been suggested that face-identity recognition may undergo qualitative changes around the time of puberty (Carey and Diamond, 1977; Carey et al., 1980) with children showing a decline in face identity recognition memory around the age of 12 years. A large scale study on nearly 500 children and adolescents, by our group, is suggestive of a similar pattern, in that improvement in face recognition memory was found to increase from 6 to 16 years of age but with a plateau in performance in the mid-pubertal years of 10–13 (Lawrence et al., 2008). Indeed, as Mancini et al. (2013) point out, the differing hormonal development of boys and girls during puberty could influence emotion recognition, suggesting that future studies should seek to explore this directly. Areas within the social brain, such as the amygdala, are populated with testosterone receptors (Filova et al., 2013) suggesting a possible mechanism by which hormonal changes during puberty might influence emotion recognition abilities. We aimed to test the hypothesis that pubertal development would influence facial emotion recognition ability. This was done in an exploratory way using a subset of the adolescent sample and, as such, should be considered as indicative rather than definitive.

The objectives of the current study were; firstly to assess the developmental trajectory of facial emotion recognition in school age children and to establish norms and developmental trends for these abilities; secondly, to ascertain whether general cognitive ability (IQ) correlates with overall emotion recognition accuracy; thirdly, to explore gender differences in these abilities; and finally, to assess whether pubertal development is related to emotion recognition accuracy.

## Materials and Methods

### Participants

Four hundred and seventy eight participants were recruited from six primary schools (age 6–11 years) and eight secondary schools (ages 11–16 years) within the London area of the UK. 20–25 males and 20–25 females were recruited within each 1-year age band. A full breakdown of gender and age group is given in **Table 1**. Schools were selected on the basis that the pupils nationally assessed levels of performance (key stage test results) were within the average range. Parents provided informed consent for their child to participate in the study. Children were excluded from testing if they had known neurological or psychological difficulties.

**Table 1:** Emotion recognition % accuracy scores for the Ekman-Friesen test of Facial Affect according to age group and gender.

Age (Years, months)		Happy(% accuracy)Mean (SD)	Surprised(% accuracy)Mean (SD)	Fearful(% accuracy)Mean (SD)	Sad(% accuracy)Mean (SD)	Disgusted(% accuracy)Mean (SD)	Angry(% accuracy)Mean (SD)
6,0–6,11	Males (*n* = 24)	83.33 (16.06)	56.67 (33.19)	39.68 (22.47)	75.83 (15.01)	30.00 (27.35)	70.42 (24.40)
	Females (*n* = 25)	95.60 (8.70)	64.80 (33.31)	40.40 (29.65)	82.80 (18.15)	34.80 (32.03)	82.40 (17.86)
7,0–7,11	Males (*n* = 19)	90.53 (15.08)	48.95 (36.65)	50.47 (30.38)	85.26 (16.11)	24.21 (27.55)	78.42 (17.08)
	Females (*n* = 24)	95.00 (7.80)	71.94 (32.58)	50.00 (25.02)	83.33 (13.41)	33.38 (29.10)	78.75 (19.41)
8,0–8,11	Males (*n* = 19)	92.63 (12.84)	76.84 (27.70)	46.84 (29.26)	72.11 (19.03)	39.47 (18.40)	69.47 (20.94)
	Females (*n* = 25)	98.40 (3.74)	72.00 (24.66)	50.22 (22.39)	79.20 (18.01)	42.40 (24.71)	73.49 (20.48)
9,0–9,11	Males (*n* = 23)	96.09 (7.83)	70.43 (34.44)	53.43 (22.20)	76.09 (16.99)	40.00 (25.94)	75.65 (13.08)
	Females (*n* = 25)	96.76 (4.83)	83.38 (16.30)	54.00 (16.33)	77.20 (13.70)	56.40 (30.40)	73.20 (19.30)
10,0–10,11	Males (*n* = 23)	97.83 (5.18)	83.48 (10.71)	49.86 (31.82)	76.96 (20.10)	42.37 (25.58)	70.87 (15.35)
	Females (*n* = 25)	96.80 (7.48)	89.60 (12.41)	52.62 (26.97)	72.80 (15.42)	62.00 (25.66)	75.20 (17.59)
11,0–11,11	Males (*n* = 27)	92.96 (8.69)	79.63 (18.70)	65.56 (19.48)	72.96 (19.38)	52.59 (26.97)	72.22 (20.06)
	Females (*n* = 24)	96.25 (6.47)	91.25 (15.69)	55.83 (27.33)	74.58 (15.03)	67.82 (26.66)	71.62 (15.17)
12,0–12,11	Males (*n* = 25)	96.40 (6.38)	80.80 (19.56)	55.20 (23.30)	78.40 (16.75)	61.60 (26.56)	67.20 (20.11)
	Females (*n* = 17)	99.41 (2.43)	82.35 (12.51)	64.31 (22.07)	80.00 (10.61)	61.18 (23.15)	78.82 (15.76)
13,0–13,11	Males (*n* = 25)	96.80 (6.90)	85.60 (13.25)	56.80 (19.09)	77.60 (17.86)	61.20 (24.55)	72.80 (16.96)
	Females (*n* = 19)	94.62 (8.64)	87.53 (14.86)	62.63 (23.30)	71.99 (13.51)	73.23 (19.88)	76.32 (17.07)
14,0–14,11	Males (*n* = 21)	99.05 (3.01)	90.95 (11.36)	59.74 (21.61)	76.19 (13.22)	74.76 (20.40)	79.05 (17.86)
	Females (*n* = 19)	96.32 (6.84)	93.16 (10.03)	68.95 (21.83)	80.53 (16.15)	82.63 (22.07)	84.21 (11.70)
15,0–15,11	Males (*n* = 15)	94.67 (9.15)	94.00 (7.37)	56.00 (23.24)	80.00 (14.64)	77.33 (21.54)	76.00 (15.02)
	Females (*n* = 9)	96.67 (5.00)	87.78 (14.81)	71.11 (23.15)	83.33 (10.00)	71.11 (23.69)	76.67 (15.00)
16,0–16,11	Males (*n* = 24)	97.50 (6.76)	84.58 (19.51)	75.42 (19.78)	75.00 (19.78)	75.42 (20.00)	76.67 (14.04)
	Females (*n* = 20)	99.00 (3.08)	88.00 (11.96)	77.50 (26.13)	74.50 (16.69)	84.50 (15.38)	78.50 (16.31)

*To assess the degree of deviance for any child, a simple *z* score can be calculated: *z* score = (child’s score – mean score for age and gender)/SD. A positive *z* score indicates the child is performing above the mean score for their age and gender, whilst a negative *z* score indicates lower than mean levels of accuracy.*

The majority of the participants were White Caucasian (*n* = 333, 69.67%). Of the remainder, 77 (16.19%) described themselves as African/Caribbean, 34 (7.11%) Indian/Pakistani, 20 (4.18%) Asian, and 14 (2.93%) described themselves as ‘Other,’ typically being of mixed-ethnicity.

At all ages, Vocabulary and Matrix reasoning *t*-scores and Full-scale IQ were within the average range for both boys and girls. Mean IQ scores ranged from 94.2 to 106.8. Independent sample *t*-tests revealed no significant differences between boys and girls at any age. The overall mean IQ score for males was 99.73 (SD 12.12, range 74–139) and 98.21 (SD 13.26, range 55–139) for females. Mean vocabulary *t*-score for males was 49.47 (SD 9.00, range 26–76) and for females 48.48 (SD 9.52, range 20–73). Mean Matrix Reasoning *t*-score for males was 49.91 (SD 8.82, range 21–72) and for females 48.76 (SD 9.78, range 20–77).

Information on pubertal status was available for a limited sub-set of the participants. The analysis looking at pubertal status as an IV was conducted on 173 participants over the age of 11 years.

### Task Descriptions

#### Facial Emotion Recognition

**FIGURE 1:**
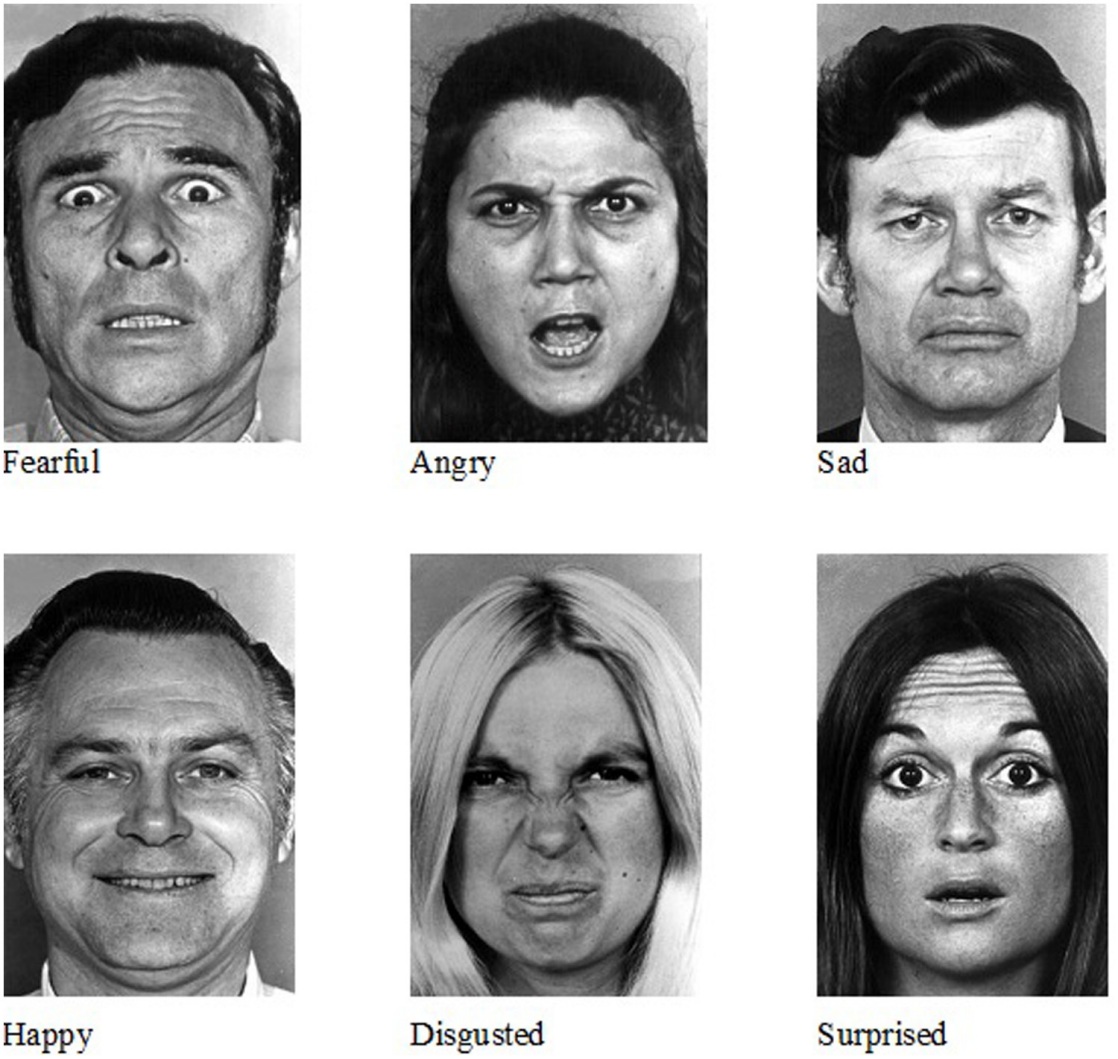
**Examples of the Ekman-Friesen Pictures of Facial Affect used in the computerized task**.

A computerized version of the Ekman-Friesen Pictures of Facial affect test (Ekman and Friesen, 1976) was developed for this study. 60 full face, uncropped images (10 of each emotion) were presented individually on a computer monitor (see **Figure 1**). Participants were required to click the mouse on the emotion label (happy, sad, angry, fearful, disgusted, and surprised – presented in this standardized order) that best described what they thought the individual was feeling. Images were presented in a single block with gender and emotion inter-mixed. The ability to read the emotional labels and respond with a mouse-click was tested in all children. Three children were unable to do this and for these cases verbal responses were given and the mouse-click made by the experimenter. Faces remained on the screen until a response was made.

#### Wechsler Abbreviated Scale of Intelligence (WASI)

Two subtests, one Verbal and one Performance, were administered according to standardized procedures (Wechsler, 1999). *t*-scores for the subscales Vocabulary and Matrix Reasoning subtests were computed and an estimated IQ score derived for each individual.

#### Pubertal Development Scale (PDS; Petersen et al., 1988)

The PDS has been reported to be a reliable measure of pubertal development. Standardization of this self-report questionnaire suggested that correlations between interview ratings and questionnaire scores had a median correlation of 0.7 (Petersen et al., 1988). Ethical permission was granted to administer the questionnaire to children of secondary school age (ages 11–16 years) but not to primary school (6–11 years) children. 16 children chose not to complete the questionnaire or provided insufficient information. Complete data were obtained for 206 children. According to scoring criteria, children were classified as pre-pubertal, beginning-pubertal, mid-pubertal, advanced-pubertal, or post-pubertal. Owing to the restricted age range assessed, very few children fell into either the pre-pubertal (*n* = 4), beginning pubertal (*n* = 22) or post-pubertal (*n* = 7) categories. Within the beginning pubertal group, the majority of participants were male. The categories with sufficient numbers to be used in the final analysis were group 3 ‘mid-pubertal’ (*n* = 73) and group 4 ‘advanced-pubertal’ (*n* = 100).

## Results

### Development of Emotion Recognition Norms

The data were inspected for outliers. One participant (an 8 year old boy) was excluded on the basis that his recognition accuracy for the Pictures of Facial Affect was at chance level (11.67%) and much lower than the next lowest score of 40% accuracy (which was achieved by three individuals with a further eight individuals achieving accuracy of between 41 and 45%).

**Table 1** shows mean accuracy scores as percentages for each gender within each age group and each emotion category. These data therefore permit any individual child or adolescent’s score to be compared with the distribution for that age-band and gender.

### IQ and Facial Emotion Recognition Abilities

Bivariate Pearson correlations were calculated for each respondent’s total emotion recognition score and their IQ. These revealed a significant relationship between emotion recognition and IQ (*r* = 0.313, *n* = 474, *p* < 0.0001) across the whole sample, indicating that tested IQ was a factor in accuracy of labeling facial emotion categories. The relationship was then examined within each age-band by separate correlations. Since 11 correlations were calculated, Bonferroni corrections were applied to the significance level (0.05/11) re-setting it to 0.005. After this correction was applied, a significant relationship between IQ and emotion recognition held at age 8 (*r* = 0.424, *n* = 44, *p* = 0.004), age 10 (*r* = 0.609, *n* = 48, *p* < 0.0001), age 13 (*r* = 0.504, *n* = 44, *p* < 0.0001) and age 14 (*r* = 0.442, *n* = 40, *p* = 0.004). Emotion recognition accuracy correlates significantly with general cognitive ability in typically developing children and adolescents; IQ was entered as a covariate into all subsequent analyses.

### Developmental Trajectory of Emotion Recognition from Facial Expression, by Gender and Age

**FIGURE 2:**
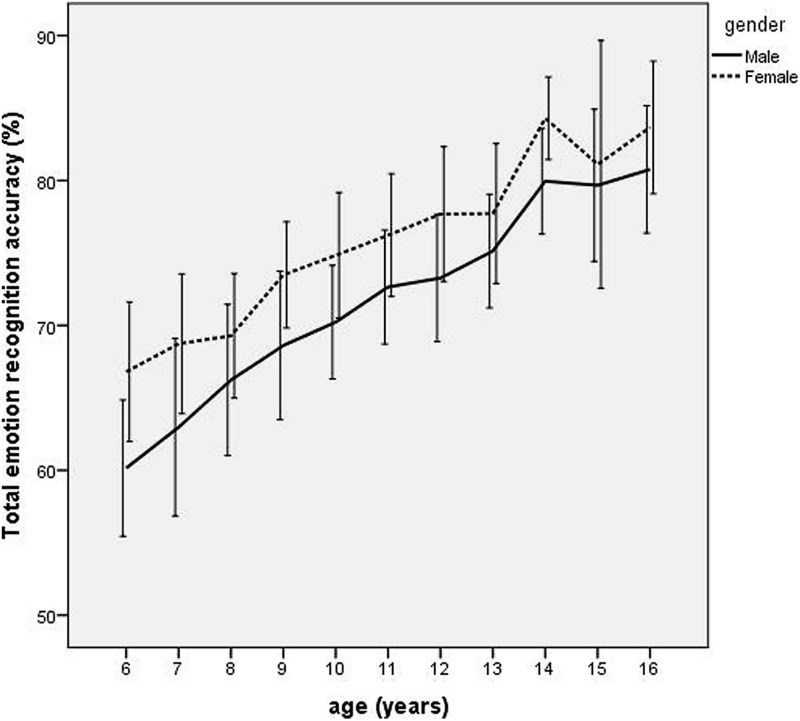
**Line graph depicting age trends in mean total emotion recognition accuracy scores for boys and girls between 6 and 16 years**. Error bars depict 95% confidence intervals.

Scores for the individual facial expressions were submitted to a repeated measures ANOVA. Percentage recognition accuracy scores for each emotion (happy, surprised, fearful, sad, disgusted, and angry) were entered as 6 levels of the repeated measure, with the 11 levels of age group (6, 7, 8, 9, 10, 11, 12, 13, 14, 15, and 16 years) and two levels of gender (male/female) as the between-subject factors and IQ as the covariate. There were significant main effects of gender [F_(1,451)_ = 24.05, *p* < 0.0001, $\eta_{p}^{2}$ = 0.05], and age [*F*_(10,451)_ = 18.39, *p* < 0.0001, $\eta_{p}^{2}$ = 0.29] qualified by a significant interaction between emotion and age group [*F*_(50,2255)_ = 7.74, *p* < 0.0001, $\eta_{p}^{2}$ = 0.15] and a significant interaction between emotion and gender [*F*_(50,2255)_ = 2.30, *p* = 0.04, $\eta_{p}^{2}$ = 0.005). As can be seen from **Figure 2**, emotion recognition accuracy increased with age, with females outperforming males at all ages.

**FIGURE 3:**
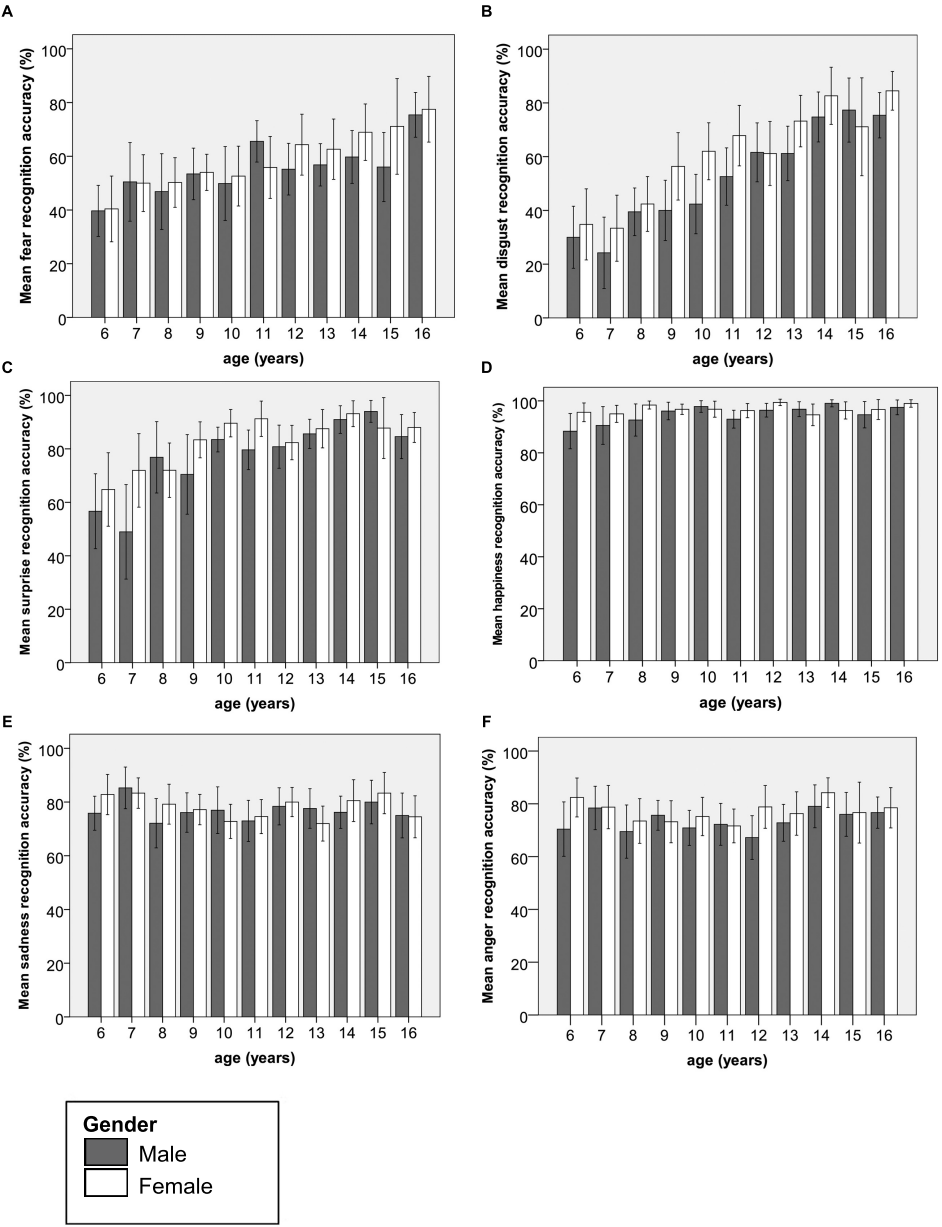
**Bar charts showing mean recognition accuracy and 95% confidence intervals for each emotion according to age and gender**. **(A)** Fear, **(B)** disgust and **(C)** surprise recognition undergo significant improvement with age. For **(D)** happiness, there is a small but significant linear improvement. For both **(E)** sadness and **(F)** anger, there is little change over time in recognition accuracy.

Recognition accuracy for each emotion was then examined using separate univariate ANOVAs to identify the source of the interactions between emotion, age group and gender. Each emotion was entered separately as the dependent variable, with age group and gender as the fixed factors and IQ as the covariate. Age group was, in each instance, subjected to polynomial contrasts in order to identify any age trends in recognition accuracy. The different age trends for each of the individual emotions, by gender, have been plotted in **Figure 3**.

#### Happiness

Happy faces were accurately named by children of all ages. At 6 years of age children could accurately name 92% of happy faces. However, there was a significant main effect of age [*F*_(10,451)_ = 2.84, *p* = 0.002, $\eta_{p}^{2}$ = 0.059] with a significant linear improvement with age identified (*p* = 0.001). Females achieved significantly higher levels of accuracy than males, although the mean difference was small [Female mean 96.75, SD 6.44; Male mean 94.86, SD 9.90; *F*_(1,451)_ = 7.67, *p* = 0.006, $\eta_{p}^{2}$ = 0.017]. There was no significant interaction between gender and age.

#### Surprise

There was a significant main effect of age for the recognition accuracy of surprised faces [*F*_(10,451)_ = 11.41, *p* < 0.0001, $\eta_{p}^{2}$ = 0.20]. Both boys and girls showed significant linear improvements with age in the ability to recognize facial expressions of surprise (difference = 29.89, *p* < 0.0001). A significant quadratic trend was also identified (difference = -15.14, *p* < 0.0001). This reflects the linear improvement up to age 10 or 11 years followed by an asymptote. Ten year olds achieve a mean accuracy score of 86.67% for surprised faces, nearly identical to the level of accuracy achieved by 16 year olds (86.14%). There was a main effect of gender, with females achieving higher recognition rates than males [Female mean 82.09, SD 22.21; Male mean 77.35, SD 26.05; *F*_(1,51)_ = 9.31, *p* = 0.002, $\eta_{p}^{2}$ = 0.02].

#### Fear

There was a significant main effect of age on the recognition accuracy of fearful faces [*F*_(10,451)_ = 7.16, *p* < 0.0001, $\eta_{p}^{2}$ = 0.14]. There was a significant linear trend in improvement of recognition accuracy for fearful faces with increasing age (difference = 28.20, *p* < 0.0001). There were no significant differences in accuracy according to gender and no interaction between age and gender.

#### Sadness

Young children were accurate at recognizing sad facial expressions. There was no effect of either age group or gender on the ability to recognize sad faces, and the interaction between these factors was non-significant. On independent samples *t*-test, there was no significant difference in the recognition of sad faces by 6 year olds (79.39%) and 16 year olds (74.77%; df 91, *t* = 1.27).

#### Disgust

There was a significant main effect of age for the recognition of disgust [*F*_(10,451)_ = 21.23, *p* < 0.0001, $\eta_{p}^{2}$ = 0.32], with a linear trend (difference = 54.99, *p* < 0.0001). Females recognized more disgusted facial expressions than males [Female mean 58.84, SD 30.50; Male mean 52.47, SD 29.56; *F*_(1,451)_ = 13.63, *p* < 0.0001, $\eta_{p}^{2}$ = 0.029). There was no interaction between age and gender.

#### Anger

There was no effect of age on the recognition of angry facial expressions. Independent samples *t*-test revealed that the scores achieved by 6 year olds (76.53%) were similar to those achieved by 16 year olds (77.50%; df 91, *t* = -0.246, n.s). A small gender effect was found for this ability [Female mean 77.01, SD 17.40; Male mean 73.31, SD 18.13; *F*_(1,451)_ = 5.81, *p* = 0.016, $\eta_{p}^{2}$ = 0.01] but there was no significant interaction between age and gender.

### Pubertal Development and Emotion Recognition

**FIGURE 4:**
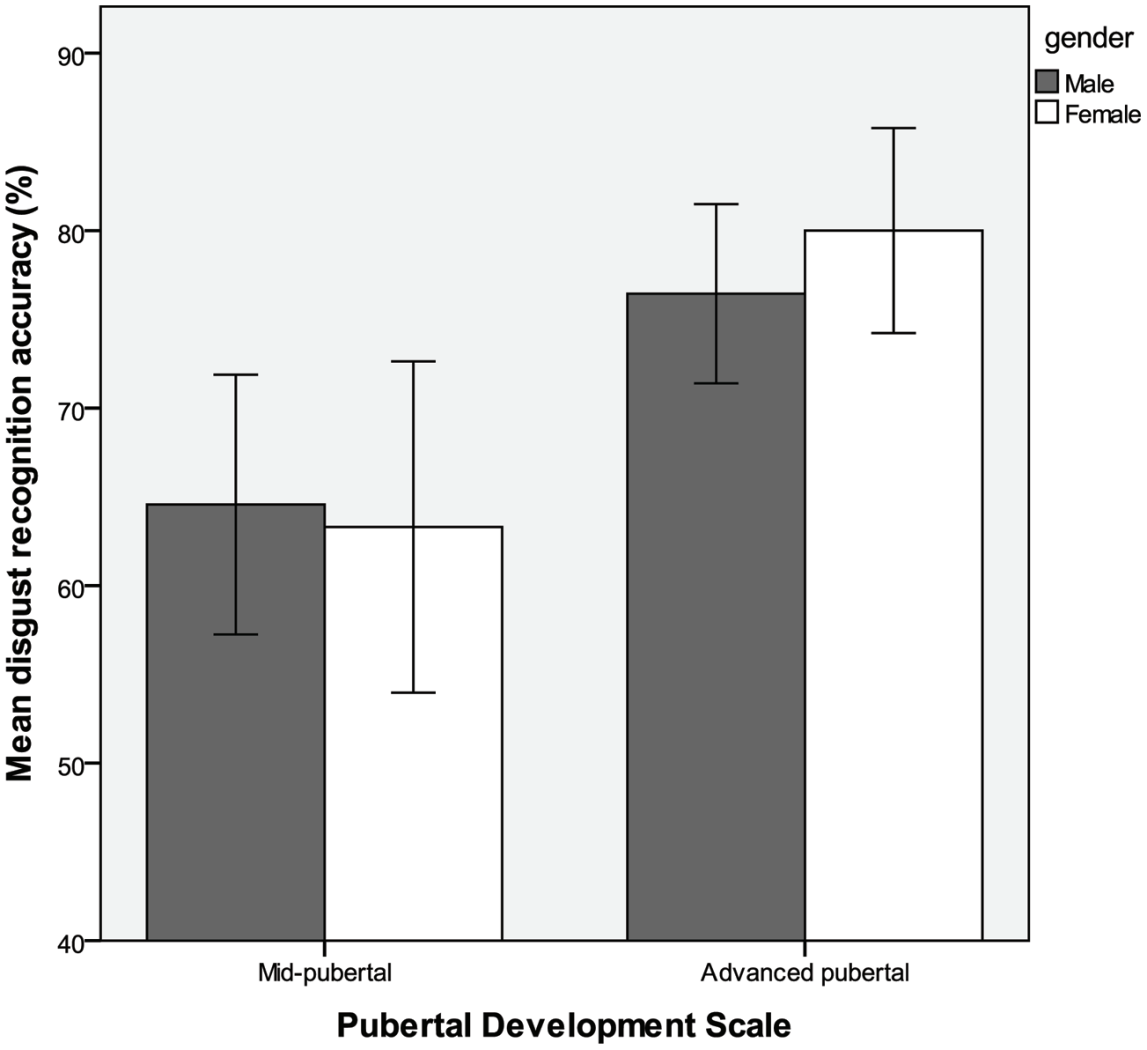
**Mean recognition accuracy and 95% confidence intervals for facial expressions of disgust in mid and advanced puberty, according to gender**.

**FIGURE 5:**
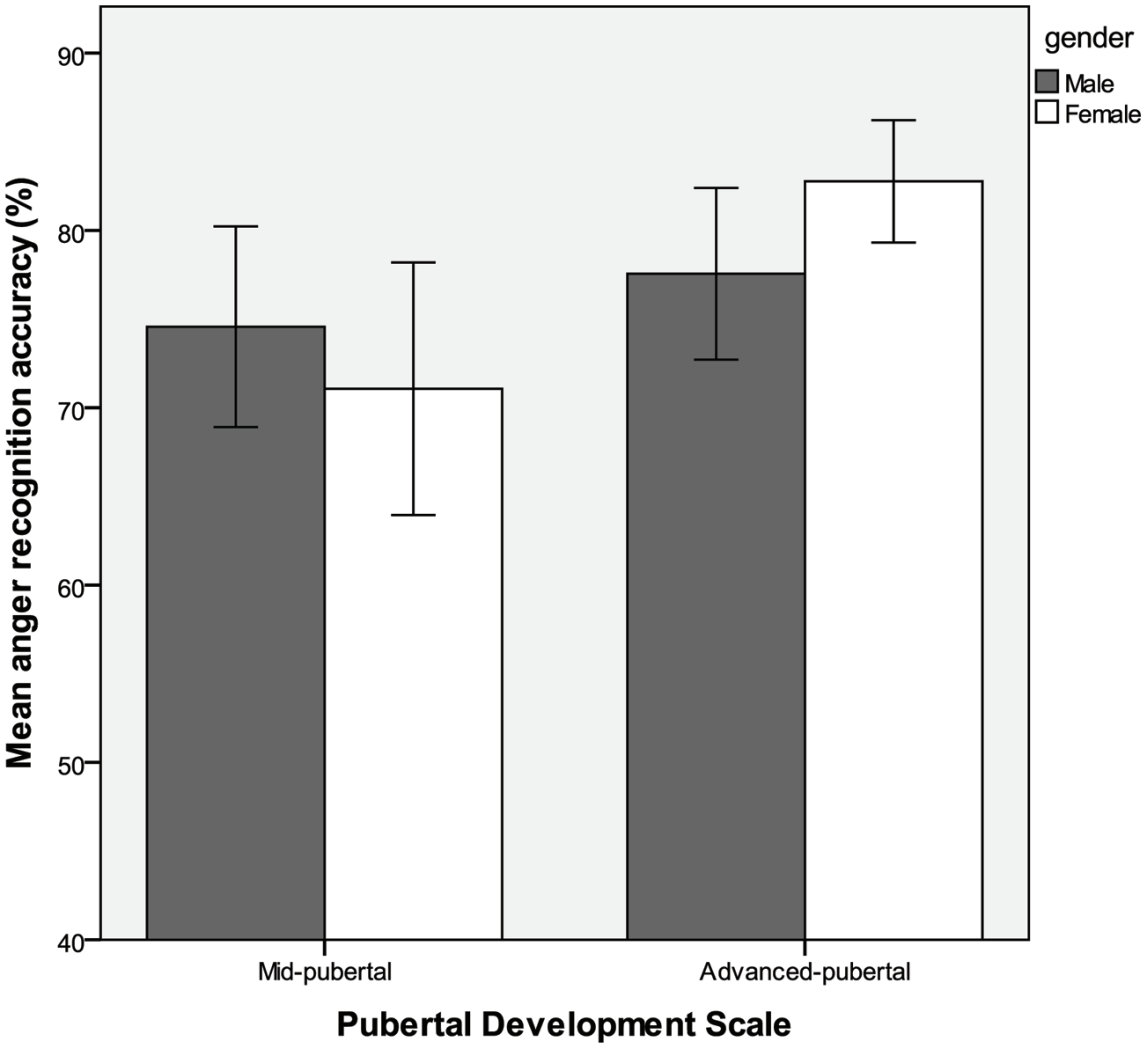
**Mean recognition accuracy and 95% confidence intervals for facial expressions of anger in mid and advanced puberty, according to gender**.

Emotion recognition accuracy was analyzed for respondents who were classified as being in the stages of mid-puberty or advanced-puberty. A multivariate analysis of covariance was performed with each of the six emotions entered as dependent variables, the two levels of pubertal development as the fixed factor, and gender, age group and IQ as covariates. Mean recognition accuracy for each of the emotions according to pubertal stage is shown in **Table 2**. There was a main effect of pubertal development on recognition accuracy for facial expressions of disgust [*F*_(1,159)_ = 7.63, *p* = 0.006, $\eta_{p}^{2}$ = 0.05) and anger [*F*_(1,159)_ = 4.10, *p* = 0.04, $\eta_{p}^{2}$ = 0.03], but no significant effect for the other facial expressions. There were no significant interactions between pubertal development and gender. Recognition of facial expressions of anger and disgust was significantly more accurate in advanced-puberty than in mid-puberty, after the effects of age, gender and IQ had been taken into account. The means and 95% confidence intervals for recognition accuracy are presented in **Figures 4** and **5**.

**Table 2:** Emotion recognition (% accuracy) scores for the Ekman-Friesen test of Facial Affect according to pubertal stage and gender.

Pubertal development		Happy(% accuracy)Mean (SD)	Surprised(% accuracy)Mean (SD)	Fearful(% accuracy)Mean (SD)	Sad(% accuracy)Mean (SD)	Disgusted(% accuracy)Mean (SD)	Angry(% accuracy)Mean (SD)
Mid-pubertal	Males (*n* = 46)	95.65 (7.20)	86.02 (14.92)	60.10 (21.00)	73.39 (15.84)	64.57 (24.65)	74.57 (19.06)
	Females (*n* = 27)	96.58 (7.54)	85.30 (16.75)	60.00 (26.75)	74.73 (13.38)	63.30 (23.58)	71.07 (18.00)
Advanced-pubertal	Males (*n* = 45)	98.22 (5.35)	87.85 (15.82)	64.22 (21.58)	78.89 (17.74)	76.44 (16.81)	77.56 (16.12)
	Females (*n* = 47)	97.23 (5.40)	89.36 (13.09)	71.70 (21.09)	78.09 (14.69)	80.00 (19.67)	82.77 (11.74)

A summary of the main effects for age, gender, and puberty is shown in **Table 3**.

**Table 3:** Overview table showing the main effects reported in this study for the individual emotions.

Significant main effects of…	Happy	Surprised	Fearful	Sad	Disgusted	Angry
Age	✓	✓	✓	×	✓	×
Gender	✓	✓	×	×	✓	✓
Puberty	×	×	×	×	✓	✓

*A ✓ refers to a significant main effect for this variable being found. A × refers to no significant main effect for this variable being found.*

## Discussion

This study establishes childhood norms for the recognition of the six basic facial expressions using the Ekman-Friesen Pictures of Facial Affect (Ekman and Friesen, 1976). Developmental trajectories for various emotions were strikingly different. We have demonstrated that the ability to recognize certain facial expressions of emotion, including fear, disgust, and surprise, improved considerably with age across childhood and adolescence. Whilst for other emotions, notably happiness, sadness, and anger, levels of recognition were very similar for 6 year olds and 16 year olds.

Most previous studies have focused on a sub-set of these emotions (Thomas et al., 2007) or on part of this developmental period (Gao and Maurer, 2010; Mancini et al., 2013). We believe this study is unique in assessing all six basic emotions in 1-year age-bands in a large sample of school age children of both genders, from the age of six to 16 years, using the same methodology (simple expression labeling) and the same materials across the age range. These normative data will be useful for appraising this skill in any individual child or adolescent, and therefore for educational or clinical diagnosis. The Ekman-Friesen Pictures of Facial Affect test is widely used for assessing emotion recognition skill in children with developmental disorders and behavioral and learning difficulties (Unoka et al., 2011; Cantalupo et al., 2013; Collin et al., 2013; Uljarevic and Hamilton, 2013; Demirel et al., 2014; Gomez-Ibanez et al., 2014), but normative data has, so far, been lacking. The results of our study allow non-normative performance to be identified. For example, individuals with autism spectrum disorders (ASDs) have been reported to have sub-optimal recognition of certain facial expressions of emotion using the Ekman-Friesen Pictures of Facial Affect in both standardized and re-developed paradigms (for example, Humphreys et al., 2007; Wright et al., 2008a). Since we have identified the scale of variation in the performance of typically developing children on this test, *z*-scores for any individual child can be compared to normative data, reducing the need for a control study. Elsewhere, we have charted developmental curves for the recognition of individual facial expressions, and developed algorithms to compute centile scores based on age, recognition score and gender (Wade et al., 2006).

Previous research has suggested children may have disproportionate difficulties recognizing particular facial expressions. It has been suggested that surprise, fear, anger and disgust may present disproportionate problems (Camras and Allison, 1985). In the current study, children were substantially worse at identifying facial expressions of fear and disgust in these adult face images than they were at recognizing any other emotional expression. Disgust and fear were the least accurately recognized emotion in 6-year olds and also showed the greatest linear improvements with age. By 16 years of age, respondents were as accurate at recognizing fearful and disgusted faces as faces depicting other emotions. In contrast, the recognition of sadness and anger showed no developmental age trends; 6 year olds were as good as 16 year olds at recognizing these emotions. A recent study by Rodger et al. (2015), consistent with the current study, found that recognition of sadness remained stable and accurate from an early age, with most other emotions showing some improvement during childhood. However, unlike our study, they also found this to be the case for expressions of surprise, which were found here to continue to show improvements until late-childhood. Variations in the methodologies used may account for this discrepancy. Since the current study used emotion labeling, whereas Rodger’s study was assessing perceptual thresholds, it seems possible that age-related increments in accuracy were related to efficiency of recognizing and/or labeling surprise, rather than any perceptual developments.

More research is needed to understand the differences between emotions and why such contrasting developmental trajectories exist. The recognition of some emotions relies more on information from the upper-face (eyes) or the lower face (mouth) or the configuration of the whole face (Calder et al., 2000; Lawrence et al., 2003a,b) but these differences seem unable to explain the differing developmental trajectories observed in childhood. Similarly, there is no obvious differentiation in neural regions recruited to process these emotions that easily explains these developmental differences.

Why might it be the case that we acquire accuracy in labeling certain emotions (such as sadness, happiness, and anger) at a much younger age than we do for other emotions (notably; fear, surprise, and disgust)? These findings would seem to be consistent with a theory put forward by Widen (2013) who has suggested that young children divide facial expressions into two categories (‘feels good’ or ‘feels bad’) and only gradually does this system of classification undergo qualitative changes, enabling children to increasingly use more specific, discrete categories. According to this theorizing, the initial distinction made within the ‘feels bad’ category is between angry and sad faces, which is in line with our finding that both these emotions were recognized accurately by the youngest participants. A further test of this idea would be to analyze incorrect responses in relation to the hypothesis: more errors would be anticipated for ‘within’ than ‘between’ the two feeling groups.

Another possible explanation for age-sensitive profiles for surprise and disgust comes from research in autism. Taylor et al. (2015) suggest that surprise and disgust may cause greatest difficulty to people with developmental impairments including ASDs and specific language impairment (SLI) because these are expressions which signal states of mind and intention. Since people with ASD often have difficulty in interpreting intentions of others (theory of mind deficits), and since tests of theory of mind show developmental progression in normally developing children, it is plausible that these expressions may be inaccurately recognized in the youngest children, and show the greatest improvement with age. A rather different interpretation for the pattern of results is that the recognition of emotion in adult faces, by children, may be susceptible to an ‘own-age bias’ effect, as has been demonstrated for face recognition (Proietti et al., 2014), and also as a factor in the effects of emotion on brain activation (Wright et al., 2008b). That is, the present findings may underestimate children’s ability to recognize certain emotions on adult faces, specifically. It is possible that those same emotions, seen on age-peer faces, may be better recognized by children. In this context it is worth noting that happiness, sadness, and anger are emotions displayed by adults to younger children which may be expected to elicit specific behaviors in the child. The ‘later-developing’ emotions (disgust, surprise, fear) may be more (age)-peer-specific in their effects.

### General Cognitive Ability and Emotion Recognition

Our study highlights the significant association between emotion recognition skills and general cognitive ability throughout childhood and adolescence, as has been previously demonstrated for facial recognition memory (Lawrence et al., 2008). This finding has been demonstrated within atypical populations; for example in children with autistic spectrum disorders and for psychiatric control children (Buitelaar et al., 1999) but our study confirms that this is also the case for typically developing children. This suggests two things. Firstly, that children with cognitive delay may have concomitant delays in their ability to accurately decode facial expressions of emotion. Secondly, when making deductions about domain specific impairments in emotion recognition accuracy within clinical populations, it is important to assess the level of general cognitive functioning in such individuals, using either a control group matched for cognitive ability or comparing scores against normative values based on mental age rather than chronological age.

### Gender Differences in Emotion Recognition

Previous research exploring sex differences in recognition accuracy for facial expressions of emotion has argued that females are more accurate decoders of emotional expressions than males (Hall, 1978; Hall et al., 1999; McClure, 2000; Montagne et al., 2005; Biele and Grabowska, 2006; Mancini et al., 2013). Our research supports and extends these findings, suggesting that during childhood and adolescence, girls are significantly more accurate than boys at recognizing facial expressions of emotion. This is consistent with the considerable popular science literature built on an assumption that females have superior empathy and emotion recognition abilities (e.g., Baron-Cohen and Wheelwright, 2004; Alaerts et al., 2011). It supports the findings of research with dynamic faces which demonstrate a female advantage throughout late-childhood (Montirosso et al., 2010). Analyzing individual emotions, it becomes clear that this pattern reflects a slight female advantage for the recognition of happiness, surprise, disgust and anger but not for the recognition of fearful or sad faces. However, this small but significant female advantage has not been replicated in all studies (e.g., Hall and Matsumoto, 2004), which may relate to differences in methodologies employed together with the sample sizes, age ranges used.

The small gender differences identified may reflect sexually dimorphic processing of facial expressions. A study by our group has suggested that men and women may call upon different functional processes for face and emotion recognition (Campbell et al., 2002). For females there was a positive correlation between the ability to recognize fearful facial expressions and face identity recognition, this correlation was absent for males. From this it was suggested that females and males may rely on different psychological processes for these tasks. The small difference in accuracy of emotion recognition between boys and girls in the current study may be reflective of subtle differences in the underlying psychological processes recruited by males and females.

Although no age by gender interactions were statistically significant in our study, other studies suggest that gender differences decrease with age. Mancini et al. (2013) report that girls had high accuracy of sadness and disgust recognition by age 8 with boys not reaching these levels until the age of 11 years, when they surpass the performance of girls. Could this male lag in the development of emotion recognition be related to anecdotal reports and observational studies that boys are more emotionally immature than girls when they start school, exhibiting different temperament characteristics to girls (Yoleri, 2014).

### Pubertal Development and Emotion Recognition

We found that anger and disgust were better recognized by respondents classified as late-pubertal compared with those who were mid-pubertal (age partialled out). These findings are similar to those reported by Thomas et al. (2007) who analyzed recognition of facial expressions morphed between neutral and anger (but did not look at recognition of disgust). They found that sensitivity to anger increased significantly between adolescence and adulthood. This is of particular interest for angry faces, as this *puberty-related* development is in stark contrast with the lack of *age-related* development of this ability in our sample. Angry and disgusted expressions could be conceived of as signaling disapproval and negative judgments of the viewer. In this sense, the finding would seem to be consistent with the observation that, as children enter adolescence, they become increasingly driven to seek the social acceptance of their peers, whilst becoming acutely sensitive to peer evaluation (Steinberg and Morris, 2001; Scherf et al., 2012). The synaptic reorganization that is evident in the adolescent brain (see Blakemore and Choudhury, 2006) may make regions dedicated to processing emotional information especially sensitive to environmental experience during this period of development. It might be hypothesized that hormonal changes during puberty differentially affect psychological processes and potentially neural circuits involved in the recognition of these facial expressions.

Due to insufficient numbers within the pre- and early-puberty groups, it was not possible to analyze the developmental trend for these recognition skills throughout the full range of the pubertal period. Future studies may benefit from collecting data across these stages of puberty in order to explore whether the linear improvement noted in our current study is preceded by any ‘dip’ in abilities in early puberty that may mirror the decline (or plateau) and subsequent improvement in facial recognition memory that has been observed in some (Carey and Diamond, 1977; Carey et al., 1980; Lawrence et al., 2008), but not all, studies. Puberty-related developments in facial emotion and facial identity recognition may be suggestive of qualitative changes in the way we process and extract meaning from faces that may in turn be under-pinned, or at the very least supported by, the structural and functional re-organization of brain circuitry recruited for such processing.

### Methodological Considerations

The Ekman faces task uses monochrome full-face photographs of adults taken during the 1970s. Even though emotions have been shown to be culturally consistent and universal (Ekman and Friesen, 1971), it is worth bearing in mind that the use of images from a different era might impact on the results of this study. Not only may there be an own-age bias (Proietti et al., 2014), but it is possible that gray-scale photographs of people wearing old- fashioned clothes, make-up and hairstyles, and who therefore seem to be from a different historical era, may elicit different responses than those of images seen to be of the respondents’ own time. Another aspect of this set of photographs is that they show very little ethnic variation, being predominantly of light-skinned Caucasian–featured individuals.

There are limitations to using the PDS, in that it relies on self-report, which may be inaccurate. Furthermore, the sensitive nature of the questions asked render it problematic to obtain ethical permission to administer, especially in younger children who are more likely to be pre-pubertal. For this reason, we have a very limited number of pre-pubertal children within our study, which makes it difficult to fully explore the development trend across all the stages of puberty. A more illuminating approach could be to adopt direct saliva testing of hormonal levels which may give, not only a more valid measurement of pubertal stage, but also be ethically acceptable to use across the entire age span of childhood and adolescence.

## Conclusion

There is burgeoning interest in the apparent deficits associated with the recognition of facial expressions among children with pervasive developmental disorders (Bolte and Poustka, 2003; Hall et al., 2003; Unoka et al., 2011; Cantalupo et al., 2013; Collin et al., 2013; Demirel et al., 2014; Gomez-Ibanez et al., 2014). The ability for clinicians to detect such impairments in an objective way relies upon the establishment of quantitative norms of the emotion recognition abilities of typically developing children. Using a well-established set of emotional face photographs (Ekman and Friesen, 1976), this study has enabled us to ascertain the normal developmental patterns of emotion recognition abilities, which are surprisingly different for different emotional expressions. Girls were more accurate than boys at recognizing some facial expressions of emotion, and pubertal maturation appeared to influence the development of the ability to recognize expressions of anger and disgust. We found considerable variance in the recognition ability of typically developing children. Such variance may in part explain why studies of emotion recognition in neurodevelopmental disorders, based on relatively small samples of clinical and control participants, often report inconsistent findings. The normative data provided in this study will aid researchers in assessing degree of impairment with more accuracy. For typically developing children, recognition of sadness, anger and happiness from facial expressions is highly accurate in early childhood. However, the ability to recognize facial expressions of fear, disgust and (to a lesser extent) surprise, matures significantly over the course of late childhood and adolescence.

If changing face and emotion recognition abilities serve as good model to understand adolescent development more generally (as suggested by Scherf et al., 2012), then researching changes in these abilities may be instrumental to developing our understanding of behavioral and mental health vulnerabilities within the teenage years. Adolescence represents a time of particular vulnerability for developing difficulties that could be seen as being associated with emotion processing or emotional regulation. For example, mood disorders such as depression and generalized anxiety disorder become increasingly prevalent in adolescence (Zuckerbrot and Jensen, 2006; Beesdo et al., 2009) and the onset of schizophrenia is often seen toward the end of the teenage years (Gogtay et al., 2011). In addition rates of antisocial behavior peak in adolescence (see Fairchild et al., 2013 for a review). Depression, anxiety, schizophrenia, and conduct disorder (which is common in those demonstrating antisocial behavior) have all been associated with deficits in facial emotion recognition accuracy (Demenescu et al., 2010; Ventura et al., 2013; Weightman et al., 2014; Sully et al., 2015). Potentially, assessing facial emotion recognition abilities in at-risk individuals might allow the detection of potential vulnerabilities, which, in turn, may have implications for intervention strategies that could provide experiential input that may encourage more appropriate emotional development during this sensitive period. As such, a fuller understanding of some of these issues could have implications for teenage mental health provision, secondary education and the remediation and legal treatment of young-offenders.

## Conflict of Interest Statement

The authors declare that the research was conducted in the absence of any commercial or financial relationships that could be construed as a potential conflict of interest.
